# Using distances between Top-n-gram and residue pairs for protein remote homology detection

**DOI:** 10.1186/1471-2105-15-S2-S3

**Published:** 2014-01-24

**Authors:** Bin Liu, Jinghao Xu, Quan Zou, Ruifeng Xu, Xiaolong Wang, Qingcai Chen

**Affiliations:** 1 School of Computer Science and Technology, Harbin Institute of Technology Shenzhen Graduate School, Shenzhen, Guangdong, China; 2 Key Laboratory of Network Oriented Intelligent Computation, Harbin Institute of Technology Shenzhen Graduate School, Shenzhen, Guangdong, China; 3 Shanghai Key Laboratory of Intelligent Information Processing, Shanghai, China; 4 School of Information Science and Technology, Xiamen University, Xiamen, Fujian, China

## Abstract

**Background:**

Protein remote homology detection is one of the central problems in bioinformatics, which is important for both basic research and practical application. Currently, discriminative methods based on Support Vector Machines (SVMs) achieve the state-of-the-art performance. Exploring feature vectors incorporating the position information of amino acids or other protein building blocks is a key step to improve the performance of the SVM-based methods.

**Results:**

Two new methods for protein remote homology detection were proposed, called SVM-DR and SVM-DT. SVM-DR is a sequence-based method, in which the feature vector representation for protein is based on the distances between residue pairs. SVM-DT is a profile-based method, which considers the distances between Top-n-gram pairs. Top-n-gram can be viewed as a profile-based building block of proteins, which is calculated from the frequency profiles. These two methods are position dependent approaches incorporating the sequence-order information of protein sequences. Various experiments were conducted on a benchmark dataset containing 54 families and 23 superfamilies. Experimental results showed that these two new methods are very promising. Compared with the position independent methods, the performance improvement is obvious. Furthermore, the proposed methods can also provide useful insights for studying the features of protein families.

**Conclusion:**

The better performance of the proposed methods demonstrates that the position dependant approaches are efficient for protein remote homology detection. Another advantage of our methods arises from the explicit feature space representation, which can be used to analyze the characteristic features of protein families. The source code of SVM-DT and SVM-DR is available at http://bioinformatics.hitsz.edu.cn/DistanceSVM/index.jsp

## Background

Protein remote homology detection is a central problem in computation biology, which refers to the detection of evolutional homology in proteins with low similarities. Through evolution, structure is more conserved than sequence. Thus, knowledge of protein structure and evolution is important for predicting the functions of proteins, which will promote protein binding study, rational drug design, and many other related fields and applications. Protein remote homology detection identifies proteins from different families, and therefore can be applied to predict structure and function of specific proteins.

Unfortunately, protein remote homology detection is still a changing problem in bioinformatics and therefore accurately and efficiently computational approaches are needed. During the past two decades, some computational methods have been proposed for protein remote homology detection, which can be mainly divided into two major categories: generative methods and discriminative algorithms. Early solutions of protein remote homology detection were generative methods, which trained a model to represent a protein family and then evaluated a query sequence according to this model. For example, BLAST [1], PSI-BLAST [2], and Hidden Markov Model (HMM) [3] searched the protein database based on a model built by both positively labeled and unlabeled proteins. Generative methods didn't perform well because they only make use of positive training samples to build the models for the prediction. Some generative methods have been improved by developing more sensitive profiles, for example, HHsearch method [4] used the hidden Markov model to calculate a novel profile. COMPASS [5] generated numerical profiles and constructed optimal profile-profile alignments. FFAS [6] was based on a new procedure for profile generation that takes into account all the relations within the family. Some online servers are available, including FORTE [7], RANKPOOP [8], webPRC [9], PHYRE [10], GenThreader [11], COMA [12], and, Bioshell [13].

The discriminative approaches mark protein sequences with a set of labels, positive if they are in the protein family and negative otherwise. These methods attempt to learn the distinction between different classes. Both positive and negative samples are used in training for these approaches. The most popular discriminative methods for remote homology detection problem are based on the Support Vector Machine (SVM) [14]. SVM learns a linear decision boundary from both positive and negative training samples to discriminate between the unseen protein sequences. A key feature of SVM is that it needs fixed length input vector. Thus some researchers have introduced different types of kernel functions and feature vectors for protein representation. Most of these kernel functions were based on sequence composition and profiles. For features based on sequence composition, some methods were based on the similarity between a protein sequence and other sequences in the training sets. For example, Fisher kernel [15] and SVM-Pairwise [16] measured the similarity from the local alignment between proteins, but the alignment score fallen into a twilight zone when the protein sequence similarity is below 35% at the amino acid level [17]. Later, these methods were improved by introducing several kernels, such as LA kernel [18], SVM-HUSTLE [19]. However these methods ignored the information from the protein structure and evolutionary information, which led to limited performance improvement. Some kernels were based on sequence features, whose feature vector were calculated from the subsequences, incorporating the protein structure information or amino acid position information. For instance, in Motif kernel [20], a protein sequence was represented in a vector space indexed by a set of motifs over a alphabet and subsequences. Spectrum Kernel [21] searched all possible subsequences of length *k *from a alphabet to form a feature map. SVM-I-sites [22] encoded structure information into the feature vectors. Mismatch kernel [23] was calculated based on shared occurrences of (*k*, *m*)-patterns in the data. LSA [24] improved the performance of building-block-based methods. SVM-N-Peptide [25] reduced the size of amino acid alphabet for increasing values of *k*. The performance of the sequence-based methods is not satisfying because these methods only use the sequence features without using the evolutionary information or 3-dimension structure. In profile-based methods, the feature vectors were extracted from profiles which contain the evolutional information. These methods showed superior performance than the sequence-based methods. This is because that a profile is much richer than an individual protein sequence in encoding information. Protein evolution involves changes of single residues, insertions and deletions of several residues, gene doubling, and gene fusion. With these changes accumulated for a long period of time, many similarities between initial and resultant protein sequences are gradually eliminated, but the corresponding proteins may still share many common features, such as similar structure and function. Profile describes this kind of evolutionary information, and therefore the profile-based kernels outperform the sequence-based kernels for protein remote homology detection. For instance, SW-PSSM [26] introduced two classes of kernel functions which were constructed from protein similarity measures by employing effective profile-to-profile scoring schemes. Profile kernel [27] used probabilistic profiles to define position-dependent mutation neighborhoods along protein sequences. A Top-n-gram-based approach [28] was proposed for protein remote homology detection. Top-n-gram can be viewed as a profile-based building block of proteins obtained by combining the most frequent amino acids in the profiles. The proteins were converted into fixed length vectors by the occurrences of each Top-n-gram and input into SVM for the prediction. Although, this method showed some improvements in predictive performance, this method completely ignores the sequence-order information. Recent studies showed that the sequence-order effects are relevant for protein remote homology detection. For example, SVM-PDT [29] incorporated the sequence-order information by considering the amino acid physicochemical properties of any two residues in a protein within a given distance. ODH [30] provided the basis distance histograms for any possible pair of *k*-mers based on distances between short oligomers, which outperformed other position independent approaches. In ACC method [31], the sequence-order information was captured by the autocross-covariance (ACC) transformation. SVM-HMMSTR [32] can capture the sequential ordering of the local structures. SVM-RQA [33] used the recurrence quantification analysis (RQA) to detect the autocorrelation patterns along the protein sequences.

Motivated by the successful of the position dependent methods, in this study, we extend the Top-n-gram-based method [28] by considering the sequence-order information, which is measured by all the possible Top-n-gram pairs within a given distance. In this approach, first, each amino acids in a protein sequence are converted into Top-n-grams based on the frequency profiles calculated from multiple sequence alignment. Second, the feature vector is calculated by the occurrences of all the Top-n-gram pairs within a given distance threshold cutoff. Finally, this feature space is input into SVM for the prediction.

## Methods

As shown by a series of publications [34-38], to develop a useful statistical prediction method or model for a biological system, one needs to engage the following procedures: (i) construct or select a valid benchmark dataset to train and test the predictor; (ii) formulate the samples with an effective mathematical expression that can truly reflect their intrinsic correlation with the target to be predicted; (iii) introduce or develop a powerful algorithm (or engine) to operate the prediction; (iv) properly perform cross-validation tests to objectively evaluate the anticipated accuracy of the predictor; (v) provide the downloadable source code or a web-server for the prediction method. Below, let us describe how to deal these procedures.

### Dataset description

#### SCOP 1.53 superfamily benchmark

We used a common benchmark [39] to evaluate the performance of our methods for protein remote homology detection, which can be downloaded at http://noble.gs.washington.edu/proj/svm-pairwise/. This benchmark has been used by many studies, which can provide good comparability with previous approaches [4,16,18,28-30,35,36]. There are 54 families and 4352 proteins selected from SCOP version 1.53. All protein sequences were extracted from the Astral database [40] and no pairwise alignments is higher than an E-value of 10^-25^. Proteins within one SCOP family were taken as positive test samples, and proteins outside the family but within the same superfamily were taken as positive training samples. Negative samples were selected from outside of the superfamily and were separated into training and test sets.

### Distance-based Top-n-gram (DT) and distance-based Residue (DR)

In this study, two approaches were proposed to convert protein sequences into feature vectors, including Distance-based Top-n-gram approach (DT) and Distance-based Residue approach (DR). First of all, we will introduce the process of the Distance-based Top-n-gram approach.

In previous study, a Top-n-gram-based method [28] was proposed for protein remote homology detection, which showed better predictive performance than some state-of-the-art methods, including SVM-LA [18], SVM-pairwise [16], and SVM-pattern [24]. Top-n-gram can be viewed as a profile-based building block of protein sequences, which contains the evolutionary information extracted from frequency profiles. Each amino acid in a protein sequence can be converted into a corresponding Top-n-gram by combining the top *n *most frequency amino acids in the corresponding column of a frequency profile, and the order of the amino acids in a Top-n-gram reflects the different importance of these amino acids during evolution. By replacing all the amino acids in a protein with their corresponding Top-n-grams, a protein sequence can be represented as a sequence of Top-n-grams instead of a sequence of amino acids. For more details of Top-n-gram, please refer to the study [28].

In order to incorporate the sequence-order information into the prediction, a Distance-based Top-n-gram (DT) approach was proposed, which extends the original Top-n-gram-based feature vector by considering the relative position information of Top-n-gram pairs in protein sequences. The proposed feature vector was calculated by counting the occurrences of all possible Top-n-gram pairs within a certain distance threshold. In this study, the Top-1-gram was selected to construct the Distance-based Top-n-gram feature vector in order to reduce the dimension of the feature vectors and reduce the computational cost. Therefore, we will introduce the proposed Distance-based Top-n-gram approach based on Top-1-gram.

Given an alphabet of Top-1-grams *Ӑ*(A, R, D, C, Q, E, H, I, G, N, L, K, M, F, P, S, T, W, Y, V), we consider the distances between all Top-1-gram pairs in a Top-1-gram sequence *S'*, which is capable of measuring the position information of the Top-1-grams sequence *S'*. Firstly, we define a distance *d *between Top-1-gram pair (*t_i_*, *t_j_*), which means that Top-1-gram *t_i _*occurs before Top-1-gram *t_j _*and the distance between *t_i _*and *t_j _*is *d*. Given a distance threshold *d_MAX_*, we set the maximum distance between Top-1-gram pair (*t_i_*, *t_j_*) as *d_MAX_*. Secondly, we count the occurrences of these distances between all Top-1-gram pairs. Thus for any distance *d *≤ *d_MAX_*, we can build a distance-based feature vector of S according to:

(1) $${\text{D}}_{d}\left(S^{\prime }\right)=\left\{ \begin{array}{l} {}[{\text{T}}_{\text{A}}^{\text{0}}\left(S^{\prime }\right),{\text{T}}_{\text{R}}^{\text{0}}\left(S^{\prime }\right),\ldots ,{\text{T}}_{\text{V}}^{\text{0}}\left(S^{\prime }\right)]\;\;\;\;\;\left(d=0\right)\\ {}[T_{\text{AA}}^{d}\left(S^{\prime }\right),T_{\text{AR}}^{d}\left(S^{\prime }\right),\ldots ,T_{\text{VV}}^{d}\left(S^{\prime }\right)]\;\;\left(1\leq d\leq d_{MAX}\right) \end{array}\right.$$

Where T^0^_i_(*S'*) is the occurrences of Top-1-gram *t_i_*, which represents the importance of each Top-1-gram occurring in *S'*; T*^d^*_ij_(*S'*) is the occurrences of Top-1-gram pair (*t_i_*, *t_j_*). The feature vector of *S' *is achieved by combining all the Top-1-gram pairs at different distances and the final feature vector can be represented as:

(2) $$F_{d_{MAX}}\text{(}S^{\prime }\text{) = [}D_{\text{0}}\text{(}S^{\prime }\text{),}D_{\text{1}}\text{(}S^{\prime }\text{),}...\text{,}D_{d_{MAX}}\text{(}S^{\prime }\text{)]}$$

The dimension of the feature vector is 20 + 20 * 20 * *d_MAX_*, where 20 is the size of the alphabet of Top-1-grams.

**Figure 1:**
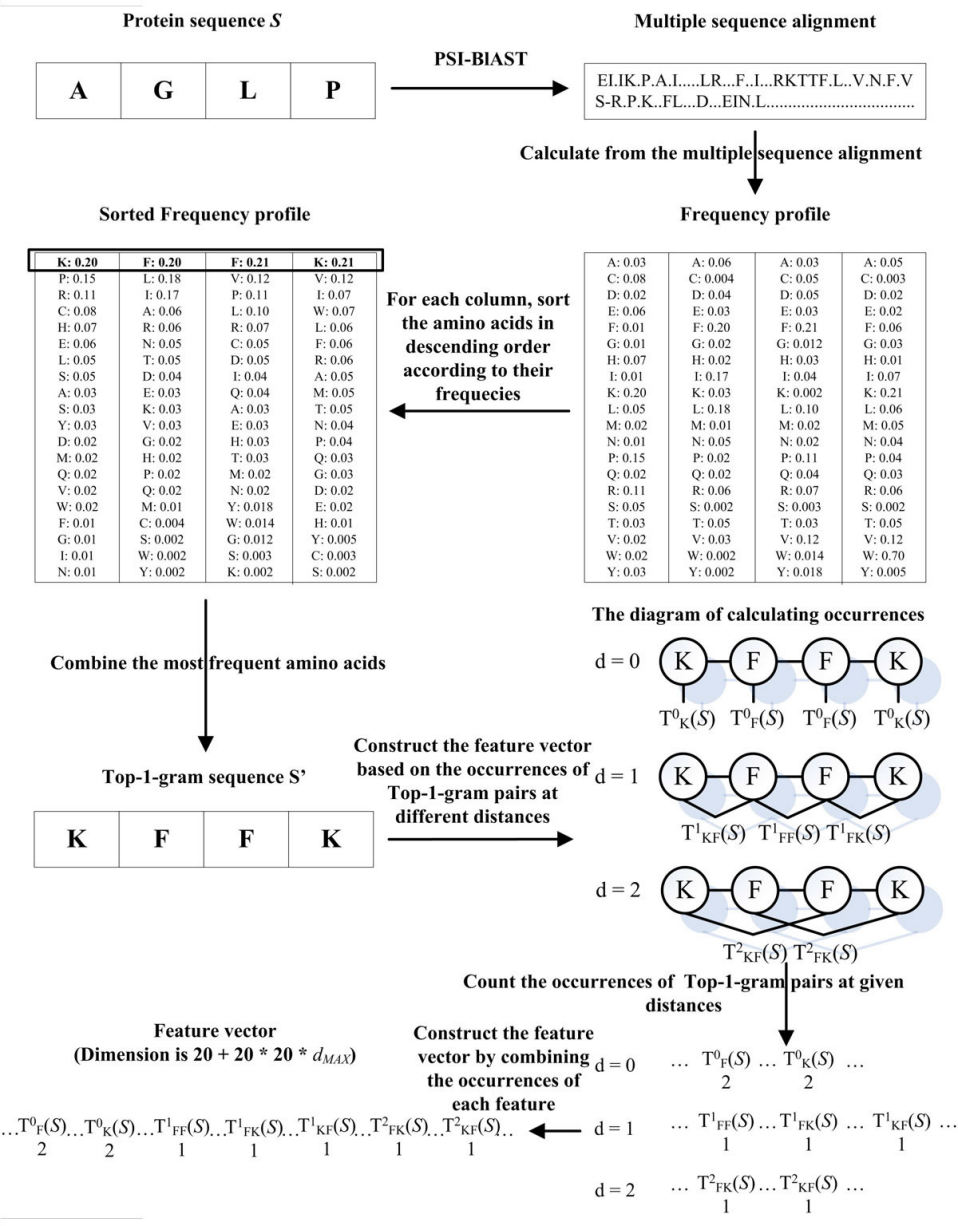
**The process of generating Distance-based Top-1-gram feature vector**. A protein *S *is input into the PSI-BLAST software to do the multiple sequence alignments against a non-redundant database, and then the frequency profile is calculated from the multiple sequence alignments. The frequencies of the 20 standard amino acids in each column of the frequency profile are sorted in descending order. Top-1-gram is the most frequent amino acid in each column of frequency profile. *S *can be represented as a sequence of Top-1-grms *S' *by combining all the obtained Top-1-grams according to their sequence order. Assuming that the distance threshold *d_MAX _*is set as 2, the feature vector is the combination of Top-1-gram pairs at distance 0, 1, and 2.

**Figure 2:**
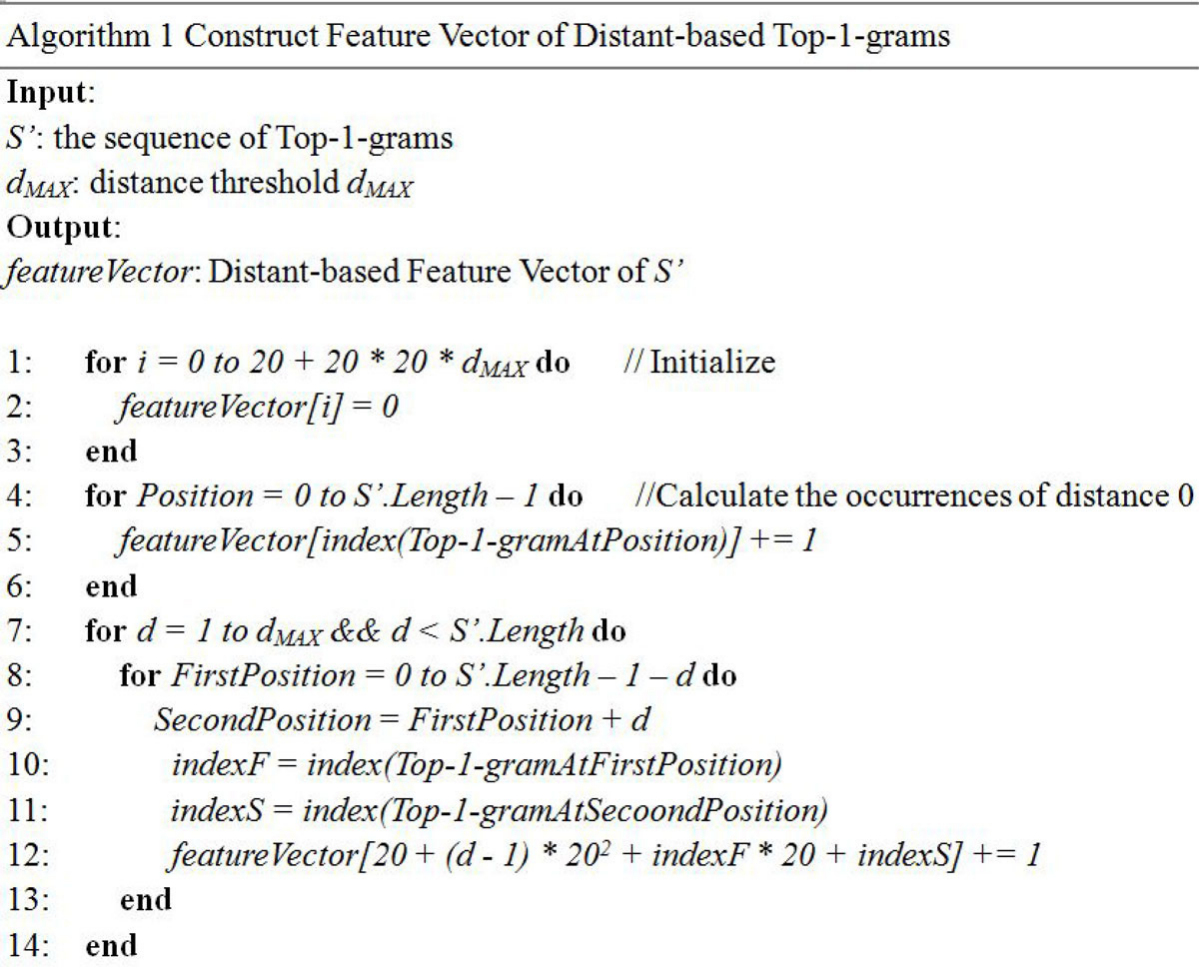
**Algorithm of construing the Distance-based Top-1-gram feature vector**. The input of this algorithm is the Top-1-gram sequence *S'*, distance threshold *d_MAX_*, and the output is the feature vector of distance-based Top-1-grams. The vector of alphabet *Index []*is the index of all the Top-1-gram in the alphabet *Ӑ*and 20 is the size of *Ӑ*, for example, index 0 indicates the first Top-1-gram in the alphabet *Ӑ*(*t_1 _*= A), and index 19 is the last Top-1-gram in the alphabet *Ӑ*(*t_19 _*= V).

In order to help the readers to further understand the process of converting a protein sequence into a feature vector, a specific example is given and shown in Figure 1. Given a protein sequence *S *= 'AGLP', each amino acid in *S *can be converted into a Top-1-gram, and therefore *S *can be represented as a sequence of Top-1-gram *S*' (KFFK). *S*' contains the evolutionary information extracted from frequency profile. If the distance threshold *d_MAX _*is set as 2, the occurrences of all Top-1-gram pairs at distance 0, 1, 2 are counted. Then the feature vector is obtained by combining the occurrences of Top-1-gram pairs at distance 0, 1, and 2. The algorithm of construing the Distance-based Top-1-gram feature vector is shown in Figure 2. The time complexity of this algorithm is O(*L*^2^), where *L *is the length of the longest protein in the dataset. The source code can be downloaded at http://bioinformatics.hitsz.edu.cn/DistanceSVM/index.jsp

The Distance-based Residue approach (DR) is similar as the Distance-based Top-1-gram approach (DT), except that the native protein sequence was directly converted into the feature vector without replacing the amino acids with Top-1-grams.

### Construction of SVM classifiers and classification

SVM learns a linear decision boundary from both positive and negative training samples to discriminate between the unseen protein sequences. A key feature of SVM is that it needs fixed length of the input vector. The proteins in the training set and test set were transformed into fixed-dimension feature vectors following the process introduced above, and then the training vectors were input into SVM to construct the classifier. The SVM gives a discriminative score for each protein in the test set, which indicates the class level of the protein. In order to have better comparability with other SVM-based methods, we employed the publicly available Gist SVM package version 2.3 (http://www.chibi.ubc.ca/gist/index.html). The SVM parameters were used by default of the Gist Package.

### Evaluation methodology

In order to evaluate the performance of SVM-based methods applied in unbalanced dataset, we applied receiver operating characteristics (ROC) score and ROC50 score to measure the performance of different methods. The ROC score is the normalized area under a curve that plots true positives against false positives for different possible thresholds for classification and the ROC50 score is the area under the ROC curve up to the first 50 false positives. The discriminative score obtained from the SVM classifier can be used to calculate the ROC score and ROC50 score.

## Results and discussion

### The impact of *d_MAX _*value on the performance of SVM-DT and SVM-DR

**Figure 3:**
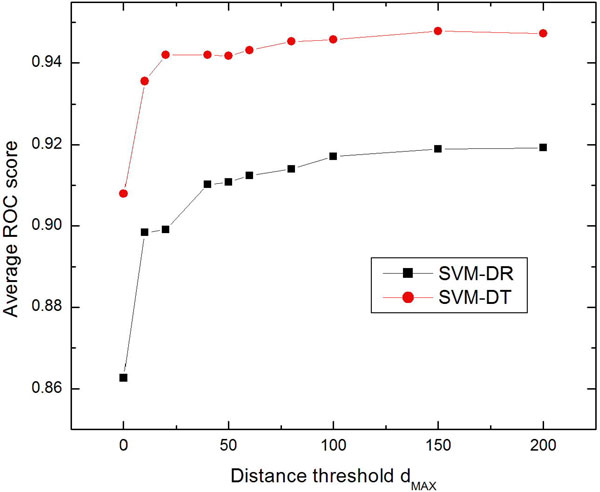
**The average ROC scores of the SVM-DR and SVM-DT with different distance threshold values of *d_MAX_***.

There is a parameter *d_MAX _*in the proposed methods (see method section for details), which would impact on the predictive performance of the proposed methods SVM-DT and SVM-DR. *d_max _*can be any integer between 0 and the length of the longest protein sequence in the dataset. Figure 3 descripts the average ROC scores of the two methods with different *d_max _*values. The performance of the two methods improves quickly with the increment of *d_max _*from 0 to 100, and the performance of both the two methods turns stable with the *d_max _*in the range of [100, 200]. These results reveal that the distance-based approaches are relevant for discrimination. In real world application, smaller *d_max _*is preferred because it leads to shorter feature vectors, and therefore less computational and space cost is needed. In this study, the *d_max _*was set as 150 considering the trade-off between performance and computational cost.

### Comparative results of previous approaches

Nine state-of-the-art protein remote homology detection methods were selected to compare with the proposed methods, including Monomer-dist [30], SVM-Top-n-gram [28], SVM-Top-n-gram-LSA [28], SVM-PDT-Profile [29], PseAACIndex-Porfile [41], SVM-LA [18], SVM-Pairwise [16], BioSVM-2L [42], and HHSearch [4]. HHSearch is a generative method, and the other eight methods as well as the proposed SVM-DR and SVM-DT are discriminative methods based on SVM. They are different in the strategies of constructing the feature vectors and kernel functions. The feature vector of Monomer-dist was based on the distances between short oligomers. SVM-Top-n-gram constructed the feature vectors by the occurrences of Top-n-grams, which can be viewed as a profile-based "building block" of proteins. SVM-Top-n-gram-LSA further improved this method by employing the Latent Semantic Analysis (LSA). SVM-PDT-Profile combined the amino acid physicochemical properties in the Amino Acid Index (AAIndex) [43] with the frequency profiles for the prediction. PseAACIndex-Porfile combined the Pseudo Amino Acid Composition (PseAAC) proposed by Chou with profile-based protein representation to convert proteins into fixed length vectors. The kernel of SVM-LA measured the similarity between a pair of proteins by taking into account all the optimal local alignment scores with gaps between all possible subsequences. BioSVM-2L constructed two-layer SVM classifiers with profile-based kernels. In SVM-Pairwise, each protein was represented as a vector of pairwise similarities to all proteins in the training set. HHSearch is one of the best protein remote homology detection methods, which employed a novel profile-based hidden Markov models.

Table 1 shows the predictive results of the two proposed methods (SVM-DT, SVM-DR) and other nine related methods. Generally speaking, profile-based methods are superior to sequence-based methods because they use the evolutionary information in profiles for protein remote homology detection. The proposed sequence-based method SVM-DR outperforms two sequence-based methods Monomer-dist, SVM-Pairwise, and is highly comparable with SVM-LA. For the profile-based methods, SVM-DT outperforms other methods except for SVM-PDT-Profile in terms of average ROC score. SVM-DT improves the SVM-Top-n-gram by considering the Top-n-gram pairs at different distances. The experimental results demonstrate that this sequence-order information is relevant for discrimination and the average ROC and ROC50 scores can be improved by 4.1% and 10.4%, respectively.

**Table 1:** Average ROC and ROC50 scores over 54 families for different methods.

Methods	ROC	ROC50	Profile	Sequence	Source
SVM-DR	0.919	0.715	No	Yes	This study
Monomer-dist	0.919	0.508	No	Yes	[30]
SVM-LA (*ß *= 0.5)	0.925	0.649	No	Yes	[18]
SVM-Pairwise	0.901	0.399	No	Yes	[16]
SVM-DT	0.948	0.800	Yes	No	This study
SVM-Top-n-gram	0.907	0.696	Yes	No	[28]
SVM-Top-n-gram-LSA	0.939	0.767	Yes	No	[28]
SVM-PDT-Profile (*ß *= 8, *n *= 2)	0.950	0.740	Yes	No	[29]
PseAACIndex-Porfile (*λ *= 5)	0.922	0.712	Yes	No	[41]
BioSVM-2L (1st+2nd layers)	0.927	0.888	Yes	No	[42]
HHSearch	0.915	0.990	Yes	No	[4]

The columns "Profile" and "Sequence" denote whether the method belongs to a class ("Yes") or not ("No"), where "Profile" indicates the method uses profile to extract the features, "Sequence" means that the method only uses the sequence composition of proteins to construct the feature vectors.

### Correlations between discriminative features and protein family

According to the above results, the proposed Distance-based Top-n-gram (SVM-DT) method showed the best performance with low computational cost when the distance threshold *d_MAX _*was taken as the value of 150. In order to further investigate the importance of the features and reveal the biological meaning of the feature space, we followed the study [29] to calculate the discriminant weight vector in the feature space. The sequence-specific weight obtained from the SVM training process can be used to calculate the discriminant weight of each feature to measure the importance of the features. Given the weight vectors of the training set with *N *samples obtained from the kernel-based training *α*= [*α*_1_, *α*_2_, *α*_3_,..., *α*_N_], the discriminant weight vector *w *in the feature space can be calculated by the following equation:

(3) $$w=M^{*}\text{a}$$

Where ***M ***is the matrix of sequence representatives. The magnitude of the element in *w *represents the discriminative power of the corresponding feature. We used the L_2_-norm of the discriminant weight vector *w *of each Top-1-gram pairs and residue pairs to measure the importance of the features.

**Figure 4:**
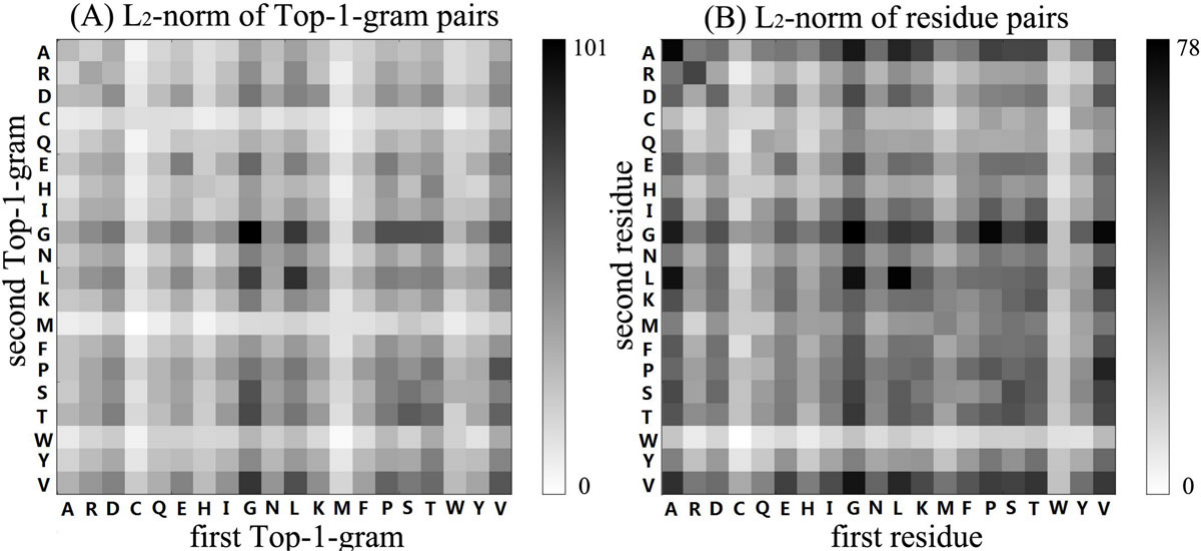
**The discriminative power (L_2_-norm) of discriminant vectors for all possible combinations of Top-1-gram pair (A) and residue pair (B) of protein family 2.5.1.3**. The amino acids are identified by their one-letter code. The amino acids labeled by x-axis and y-axis in figure(A) indicate the first Top-1-gram and the second Top-1-gram in Top-1-gram pairs of SVM-DT, respectively; the amino acids labeled by x-axis and y-axis in figure (B) indicate the first residue and the second residue in residue pairs of SVM-DR, respectively. The adjacent color bar shows the mapping of L_2_-norm values.

Family 2.5.1.3 was selected as a target family for the feature analysis. The predictive results of SVM-DT on this family are obviously higher than those of the SVM-DR (0.993 VS 0.844 in terms of average ROC score). The L_2_-norm of 400 Top-1-gram pairs and residue pairs for these two methods are depicted in Figure 4. According to the figure, interestingly, the top two most discriminative pairs are (G, G), (L, L) for both of the two methods (the two darkest spots in each subfigure of Figure 4), which indicates the importance of amino acid G (Glycine) and L (Leucine). The strong discriminative power of Top-1-gram pair (G, G) on protein family 2.5.1.3 (Multidomain cupredoxins) is not surprising, because highly conserved sequence PHGGGWGQ are abundant in cupredoxins [44], and Top-1-gram pair (G, G) can capture this sequence pattern, which would be the reason for better performance of SVM-DT on this protein family.

**Figure 5:**
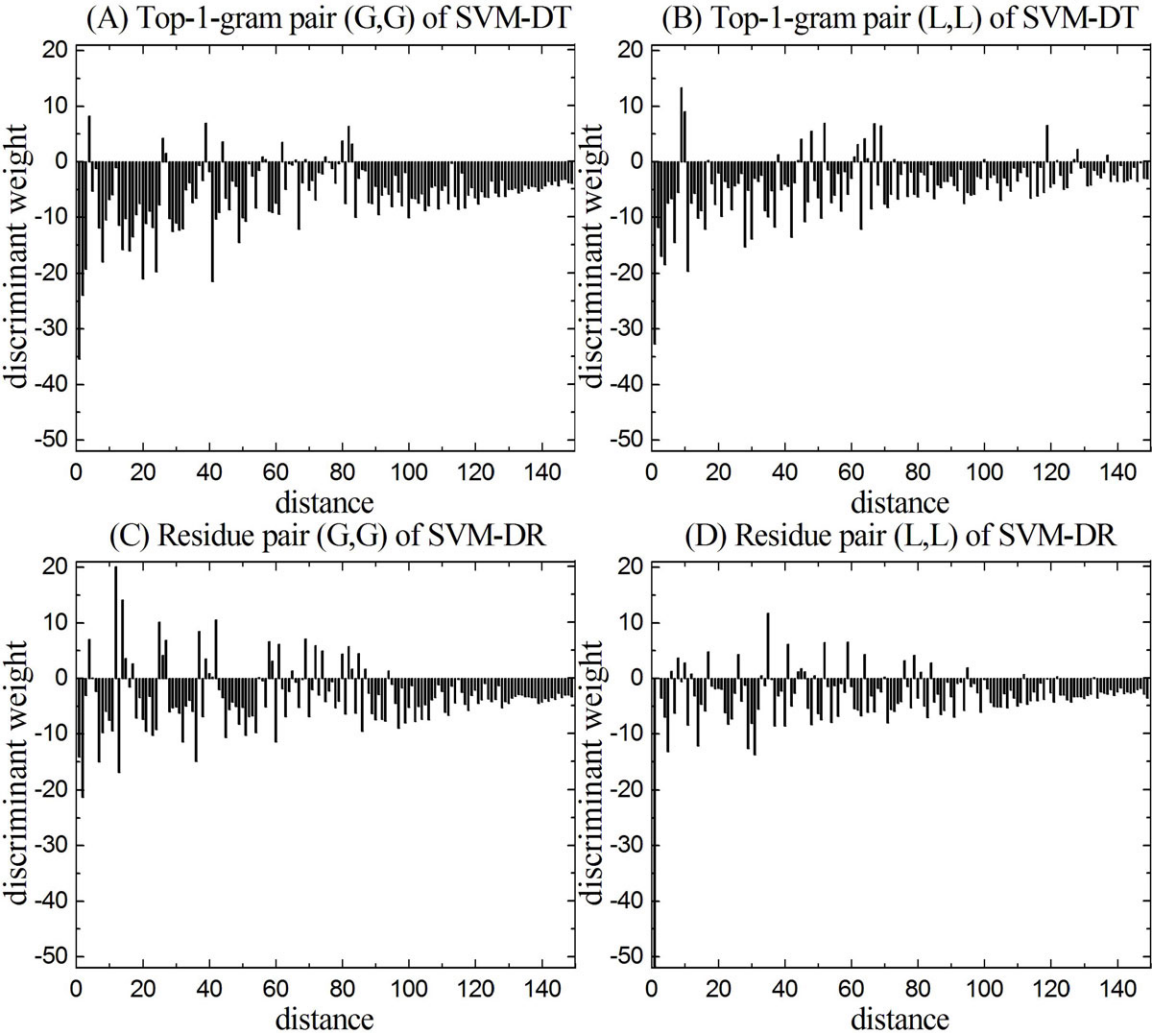
**The discriminant weights of the most discriminative Top-1-gram pairs (G, G) and (L, L) of SVM-DT for family 2.5.1.3 are shown in figure (A) and (B), respectively; the discriminant weights of the most discriminative residue pairs (G, G) and (L, L) of SVM-DR for family 2.5.1.3 are shown in figure (C) and (D), respectively**.

Figure 5(A) and 5(B) show the discriminant weights of the top two most important Top-1-gram pairs (G, G) and (L, L) of the SVM-DT on family 2.5.1.3, and the discriminant weights of the top two most important residue pairs (G, G) and (L, L) of the SVM-DR are shown in Figure 2(C) and 2(D), respectively. As indicated by the above results, short distances are more important for discrimination than longer distances, which coincides with the ladder-shaped structure of discriminant values for distances. These results demonstrate that using the Top-1-gram pairs with distances shorter than a given distance threshold *d_MAX _*to construct the feature vector is an efficient strategy to reduce computational cost, because shorter distances are more important than longer distances for protein remote homology detection. Figure 5(A) shows the discriminant weight of pair (G, G) of SVM-DT and the magnitude of zero-distance shows the importance of Glycine frequency for discrimination. Most of the distances between Top-1-gram pairs (G, G) show positive discriminative power, while only a few distances show negative discriminative power, such as the distances 3, 25, 26. Figure 5(B) shows the discriminant weight of pair (L, L) of SVM-DT, which shows similar patterns. Note that the Top-1-gram pairs with zero-distance always show higher discriminative power than other distance values for both of the two features, indicating the local structure, especial the subsequence of proteins are very important for protein remote homology detection. Figure 5(C) and 5(D) show the discriminant weights of the top two most important residue pairs of the SVM-DR on family 2.5.1.3 after SVM training process. These two subfigures also show similar ladder-shaped structure, but there are more features show positive discriminative power than those in the SVM-DT as shown in Figure 5(A) and 5(B).

## Conclusion

In this study, we proposed two methods SVM-DT and SVM-DR for protein remote homology detection, in which the feature vectors were constructed based on the occurrences of Top-n-gram pairs or residue pairs at distances shorter than a distance threshold *d_MAX_*. These approaches can be viewed as position dependant methods that incorporate the sequence-order information. SVM-DR is a sequence-based method, its advantage is that it doesn't need time consuming multiple sequence alignment step. SVM-DT is a profile-based method, which achieves more accurately predictive performance but higher computational cost is required due to the generation of Top-n-grams. Recently, position dependant methods have been attracted much attention. Remote homology proteins share low sequence similarity, and therefore, structure information is a key to improve the predictive performance. These position dependant methods partly incorporate the structure information by considering the relative orders of residues or other building blocks of proteins occurring in protein sequences, such as Monomer-dist proposed by Linger *et al *[30]. This method used the distances between short oligomers to produce the feature vectors, which gave rise to very high-dimensional feature vectors. In contract, SVM-DR efficiently reduced the dimension of feature vectors by only considering the residue pairs at distances shorter than a distance threshold *d_MAX_*. SVM-DT further improved SVM-DR by using Top-n-grams to replace the residues in proteins and produced feature vectors based on Top-n-gram distances. This profile-based method used the evolutionary information in profiles and therefore showed better performance than the sequence-based methods and the position independent methods, such as SVM-Top-n-gram [28], indicating that the distance-based approaches are relevant for discrimination. Recent studies showed that besides sequence and profile information, other features describing the physicochemical properties of amino acids can accurately detect the protein homologies, such as the amino acid index (AAIndex) [29,41]. We are looking forward to incorporating these features into the proposed distance-based framework and exploring new mathematical and statistical models for the representation of protein sequences.

## Competing interests

The authors declare that they have no competing interests.

## Authors' contributions

BL conceived of the study and carried out the protein remote homology detection study, participated in designing the study, coding the experiments, drafting the manuscript and performing the statistical analysis. JHX participated in coding the experiments and drafting the manuscript. RFX, QZ, XLW, QCC participated in performing the statistical analysis. All authors read and approved the final manuscript.

## Declarations

The publication costs for this article were funded by the corresponding author.

This article has been published as part of *BMC Bioinformatics *Volume 15 Supplement 2, 2014: Selected articles from the Twelfth Asia Pacific Bioinformatics Conference (APBC 2014): Bioinformatics. The full contents of the supplement are available online at http://www.biomedcentral.com/bmcbioinformatics/supplements/15/S2.
